# Letters and Answers to Correspondents

**Published:** 1872-10

**Authors:** 


### Our Correspondence.

### LETTERS EXPOSING HUMBUGS AND SWINDLERS,

WITH OTHER COMMUNICATIONS, OF INTEREST TO THE PEOPLE.

MILAN, TENN., Aug. 20, 1872.

DR. T. S. UP DE GRAFF:

*Dear Sir.*—Permit me to call your attention to an article in the January number of the Bistoury, on page 300, taken from the *Philadelphia Medical Times*, and written by Dr. J. E. Reeves, on Cholera Infantum. In the latter prescription he says: "Sig: *One every three or four hours.*"

He certainly has omitted a very important part of his directions; he evidently meant or intended to divide his prescription into 10 or 12 powders. I call his attention to this, simply because I think he has overlooked it.

*Five grains* of Dover's Powder would certainly produce very unpleasant effects upon a child; and is calculated to lead the young practitioner into serious results. I have used his prescription with very beneficial results, but *always divided it in twelve powders.* I think he surely overlooked what he intended to do—the division of his prescription into doses.

I hope he will pardon me for calling his attention to what I think is an oversight in him.

Yours, respectfully,

A. A. DAVIDSON, M. D.

—The above letter explains itself. The error referred to is a serious one, and should be corrected. Subscribers who file away their numbers of the Bistoury, should write in the margin of the page, and opposite the formula referred to, "Divide into twelve powders."

---

BLOSSBURG, PA., Aug. 25, 1872.

DR. UP DE GRAFF:

*Dear Sir.*—There is a chap stopping here who calls himself "Dr. G. D. Crandal, Oculist, Aurist, and General Surgeon, of *Up de Graff's* Eye and Ear Infirmary and City Hospital." Do you know anything of him, and has he any authority to use your name? J. R.

—He is a swindler and imposter. We do not send out agents or quack, traveling doctors to represent us. He has never been under our instruction nor in our employ. We know nothing of him.

———

CORNING, N. Y., Sept. 1, 1872.

EDITOR BISTOURY:

I have been treated by "Dr. John E. Smith and Associates," the traveling mountebanks, who visit our place occasionally; have paid them considerable money, swallowed their "University Medicines," and now find myself as bad, if not worse off, than I was before. They still ask me to continue the "treatment," and to correspond with them at Cleveland, Ohio, promising me a cure eventually, if I persist in buying their medicines. Now, what would you do in such a matter? Is there no way of punishing such swindlers? B.

Well, we do not know *what* you can do in the matter, unless you apply to the next traveling doctor that comes in your way. Perhaps you had better apply to a clairvoyant and be "doctored for a tape-worm;" that seems to be the prevailing delusion just now.

———

ROCHESTER, July 20, 1872.

DR. UP DE GRAFF:

Dr. ——, the traveling oculist, operated upon my eye for cataract. He said he could "absorb" it by putting a needle in my eye once a month. He has done so, but it does not get any better. He now wants to push it down, out of the pupil, with a needle, and says that will cure it.—Would you advise such an operation? I have paid him fifty dollars, and am anxious to get the value of my money if I can. L. S. R.

—If you desire to lose your eye as well as your money, we would advise you to continue the services of your traveling oculist. That is not the sort of an operation that skillful ophthalmic surgeons recommend now-a-days, but just such a one as we would expect from the talent (?) you have employed. Employ the tape-worm woman—you are a fit subject.

———

UTICA, N. Y., Aug. 25, 1872.

EDITOR BISTOURY:

Is there any virtue in "wild tea," for the cure of cancer and scrofula? J. Henry Smith, of 83 Fifth Avenue, Pittsburgh, Pa., has sent a package to my mother for trial, and she has a desire to send ten dollars for a pound of it. I thought I would first inquire of you about it.

Respectfully, P. M. S.

—O, pshaw! We have exposed that swindle two or three times in previous numbers of the Bistoury. But as you are a new subscriber, we will tell you confidentially, that J. Henry Smith and his wild tea, are abominable frauds.

———

SHEFFIELD, ONTARIO, June 11, 1872.

T. S. UP DE GRAFF, M. D.

Do you know anything about Dr. R. Hunter, of Cincinnati, Ohio? He treats the lungs by inhalation. We have seen his advertisement and thought of patronizing him. J. L. D.

—We know nothing of him, but answer upon general principles. If he advertises in the public prints, to cure consumption by means of any particular apparatus or system, he is promising to perform that which he knows to be impossible, therefore, is a swindler.

———

OLEAN, N. Y., Aug. 27, 1872.

DR. UP DE GRAFF:

Will you be kind enough to inform me through the Bistoury, or by letter, what course I had better pursue with my eyes. I have been compelled to use my eyes much by lamplight, sewing upon all sorts of fabrics. Lately my eyes blur and burn after sewing awhile, so that I am compelled to throw down my work and bathe them in cold water for relief. I notice dark spots, which seem to be floating before my eyes, and the white portion of the eye, at times, becomes blood-shot. What can I do to relieve them? * * * * MRS. H.

—You should at once see the necessity for discontinuing the use of your eyes in sewing and reading. But we doubt whether we can advise any treatment that you could safely conduct yourself. Better apply to some ophthalmic surgeon for an ophthalmoscopic examination of your eyes. Your symptoms predict early blindness, unless relief is speedily had. Do not trifle with nostrums, but secure reliable advice, without delay.

———

WELLSBORO, PA., Aug. 20, 1872.

DR. UP DE GRAFF:

My little boy has a soft, elastic swelling, resembling a "wind gall" of the horse, situated near the knee-pan of his right knee. It is not painful, neither is it sore to the touch. It seems to be growing. What should be done for it?

B.

—It is a synovial bursa, caused by an escape of the synovial fluid of the joint, under the skin. It should be treated by pressure. An elastic knee-band, properly fitted and applied, would remedy the difficulty.

READING, PA., Sept. 2, 1872.

DR. UP DE GRAFF:

Can club-foot be cured in a child six months old, without an operation? J. P.

—Some forms of club-foot can be cured by means of a suitable apparatus, and without cutting. We cannot advise you understandingly, without seeing the case. Have it attended to as early as possible; particularly before the child is allowed to stand upon its foot, and so rendering the malformation worse.

HIESVILLE, KY., July 30, 1872.

DR. UP DE GRAFF:

My son has an ugly looking sore upon his leg, as large as a silver dollar. We have tried everything we could hear of, and have had it treated by physicians, but without being able to heal it. Can you advise us what to do with it?

A. C.

—Apply to an intelligent physician. Have the boy given proper tonics of quinine and iron. Apply carbolic acid to the ulcer, for a week or ten days, or as long as may be necessary to cause the granulations to assume a healthy appearance, then have it skin-grafted. Any surgeon "up with the times," can so heal it for you.

HANNIBAL, MO., July 30, 1872.

DR. UP DE GRAFF:

My employment often subjects me to injuries, so that I frequently get bruises upon my body and occasionally a "black eye." What can be done to prevent the blood from settling under the skin and changing from black to blue, yellow, green, and in fact all sorts of diabolical colors? F. M.

—Apply cloths, wrung out of hot water, frequently, during the day. Afterward, tincture of arnica. The hot water will prevent the blood from coagulating, enabling it to become absorbed within twenty-four hours.

EAST STOUGHTON, MASS., July 25, 1872.

EDITOR BISTOURY:

Having read your replies to various correspondents suffering from all sorts of maladies, I am emboldened to seek advice through the same channel. My wife, aged 40, has recently complained of considerable pain in her back, has swollen ankles, and sometimes I notice a puffiness about the eye-lids. She has a capricious appetite, and an occasional diarrhœa, and complains of great and increasing prostration. Her urine is very scanty, and has a very thick, whitish deposit. She has been treated by several physicians with tonics, &c., but she gradually grows worse. What do you imagine her trouble to be, and what course should we pursue with a view to restoring her health?—We will wait with patience for a reply through the Bistoury, not expecting you to take time to reply by letter. M. J. R.

—Taking your account of the case solely as a guide, we should pronounce it to be Bright's disease of the kidneys. Your family physician should prescribe the remedies, as no successful treatment could be conducted by other than a physician who could see her daily. Use no patent medicines or advertised nostrums—they are worse than useless.

